# Longitudinal analysis of the strengths and difficulties questionnaire scores of the Millennium Cohort Study children in England using *M*‐quantile random‐effects regression

**DOI:** 10.1111/rssa.12126

**Published:** 2015-07-01

**Authors:** Nikos Tzavidis, Nicola Salvati, Timo Schmid, Eirini Flouri, Emily Midouhas

**Affiliations:** ^1^ University of SouthamptonUK; ^2^ Università di PisaItaly; ^3^ Freie Universität BerlinGermany; ^4^ University College LondonUK

**Keywords:** Influence function, Multilevel modelling, Quantile regression, Repeated measures, Robust estimation

## Abstract

Multilevel modelling is a popular approach for longitudinal data analysis. Statistical models conventionally target a parameter at the centre of a distribution. However, when the distribution of the data is asymmetric, modelling other location parameters, e.g. percentiles, may be more informative. We present a new approach, *M*‐quantile random‐effects regression, for modelling multilevel data. The proposed method is used for modelling location parameters of the distribution of the strengths and difficulties questionnaire scores of children in England who participate in the Millennium Cohort Study. Quantile mixed models are also considered. The analyses offer insights to child psychologists about the differential effects of risk factors on children's outcomes.

## 1 Introduction

Early exposure to family poverty, stressful life events and neighbourhood disadvantage constitute risks for children's emotional and behavioural adjustment (Bradley and Corwyn, 2002; Flouri *et al*., 2010; Goodnight *et al*., 2012). The cumulative risk literature indicates that, as risk factors accumulate, children's emotional (internalizing) and behavioural (externalizing) problems increase (Trentacosta *et al*., 2008). Internalizing behaviours are typified by inward symptoms such as being withdrawn, fearful or anxious. Externalizing behaviours are outward and may be described as aggressive, non‐compliant, impulsive or fidgety. One widely used measure of children's emotional and behavioural problems in psychological research is the strengths and difficulties questionnaire (SDQ) (Goodman, 1997). The SDQ score is the sum of the main caregiver's responses to a series of items that describe children's internalizing and externalizing problems. The 25‐item SDQ comprises five domains measured with five items each, namely emotional symptoms (e.g. ‘many fears; easily scared’), peer problems (e.g. ‘gets on better with adults than with other children’), conduct problems (e.g. ‘often lies or cheats’), hyperactivity (e.g. ‘restless; overactive; cannot stay still for long’) and prosocial behaviour (e.g. ‘shares readily with other children’). For each item, 0 is given if the response is not true, 1 if somewhat true and 2 if certainly true. The internalizing SDQ score is the sum of responses to the five emotional symptoms items and the five peer problems items. Therefore, the range of internalizing scores is 0–20. The externalizing score is the sum of responses to the five conduct problems items and the five hyperactivity items (also ranging from 0 to 20). The SDQ is a valid and reliable measure of children's emotional, social and behavioural difficulties. More information can be found at www.sdqinfo.com. Recent literature (Flouri *et al*., 2014a; Midouhas *et al*., 2014) has systematically examined the effects of neighbourhood and family risk factors on the Millennium Cohort Study (MCS) children's trajectories of SDQ scores. Owing to the longitudinal structure of the cohort data, these studies make extensive use of multilevel models, which are also referred to as random‐effects or mixed models (Steele, 2008).

**Figure 1:**
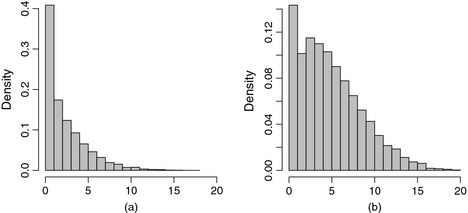
Histogram for (a) SDQ internalizing and (b) externalizing problems scores

Conventionally, random‐effects models target the expected value of the conditional distribution of the outcome given a set of covariates. When the distribution of the outcome is asymmetric, modelling other location parameters, e.g. percentiles of the conditional distribution, may offer a more complete picture compared with a model that describes only the centre of a distribution. The distribution of SDQ outcomes is typically asymmetric. This is illustrated in Fig. 1. To the best of our knowledge, the above‐mentioned studies that analyse the SDQ scores fail to recognize the asymmetric shape of the SDQ distributions. Modelling the conditional mean may therefore not offer the best summary. An alternative measure of the centre of a distribution, such as the median, may be more appropriate in this case. To illustrate, it is possible that the effect of certain risk factors on the SDQ scores is not the same across the distribution of SDQ scores. For example, maternal depression consistently has a strong association with mean child adjustment (Kiernan and Huerta, 2008) but may have a more pronounced effect at the top end where children display a high, perhaps abnormal, level of adjustment problems than at the bottom end of the distribution. Quantifying the effects of risks factors in this way can offer useful insights to child psychologists.

The idea of modelling location parameters has a long history in statistics. The seminal paper by Koenker and Bassett (1978) is usually regarded as the first detailed development of quantile regression, which is a generalization of median regression. Extensions of quantile regression for modelling multilevel‐type data have been considered by several researchers including Koenker (2004) and Geraci and Bottai (2007, 2014). Koenker (2004) proposed the use of penalized quantile regression for longitudinal data, where the penalization is aimed at the shrinkage of individual effects. Geraci and Bottai (2007, 2014) proposed a linear quantile mixed model (LQMM) estimated by using maximum likelihood and the link between quantile regression and the asymmetric Laplace distribution. The distribution of the random effects is assumed to be either Gaussian or Laplace, with the latter offering robustness properties. The use of the asymmetric Laplace distribution by Geraci and Bottai (2007, 2014) is mainly for convenience as it provides a parametric link between maximum likelihood estimation and minimizing the sum of absolute deviations. Inference for the model parameters is performed by using a bootstrap based on resampling the sample data. Estimation and inference are facilitated by the lqmm function in R (R Development Core Team, 2010).

There are, however, alternatives to quantile regression, such as *M*‐quantile (MQ) regression (Breckling and Chambers, 1988; Chambers and Tzavidis, 2006), which is a quantile‐like generalization of regression based on influence functions (*M*‐regression), and expectile regression (Newey and Powell, 1987), which is a quantile‐like generalization of mean regression. Currently, the available MQ regression models assume independent observations and hence do not allow for the analysis of multilevel‐type data. Although approaches to *M*‐estimation in random‐effects models have been proposed by a series of researchers (Huggins, 1993; Richardson and Welsh, 1995), the focus of their work is on modelling a location parameter at the centre of the conditional distribution rather than the entire conditional distribution. This paper proposes an extension of MQ regression to *M*‐quantile random‐effects (MQRE) regression.

In particular, from a methodological point of view the present paper proposes a novel approach for modelling location parameters by using MQRE regression. The methodology proposed can be viewed as an alternative to the LQMM that was proposed by Geraci and Bottai (2014), although the two models target different population parameters. Furthermore, the LQMM allows for the specification of both random intercepts and random coefficients. In contrast, the MQRE approach that we propose in this paper can currently accommodate only random intercepts. From an applied point of view, the present paper extends recent studies on the effect of risk factors on the SDQ scores of children in England by using MCS data. The paper further presents a range of existing and newly proposed modelling tools which are available to the prospective data analyst for modelling a general set of location parameters of a distribution via quantile and MQRE regression.

Why consider MQs when one key advantage of quantile regression is the more intuitive interpretations? Although not the same, both quantile and MQ models target essentially the same part of the distribution of interest. As we shall see in this paper, one of the main advantages of *M*‐estimation is that it easily allows for robust estimation of both fixed and random effects. Furthermore, because MQs are based on the use of influence functions, we can trade robustness for efficiency in inference by selecting the tuning constant of the influence function. Finally, the use of a range of continuous influence function, instead of only an absolute value of 1 as in quantile regression, can potentially offer computational stability.

The structure of the paper is as follows. In Section 2 we describe the data. Section 3 reviews random‐effects regression and focuses specifically on robust estimation of model parameters. In Section 4 we review MQ regression, present the proposed MQRE regression and discuss estimation and inference. In Section 5 we present the results from the application of MQRE and quantile random‐effects regression (Geraci and Bottai, 2014) to the SDQ scores of the MCS children in England. In Section 6 we empirically evaluate the properties of MQRE regression by using Monte Carlo simulation studies under a range of data‐generating mechanisms. Finally, in Section 7 we conclude the paper with some final remarks.

## 2 The data

The MCS (www.cls.ioe.ac.uk/mcs) is a longitudinal survey drawing its sample from all births in the UK over a year, from September 1st, 2000. The MCS was designed to overrepresent areas with high proportions of ethnic minorities in England, areas of high child poverty and the three smaller UK countries. Sweep 1 took place when the children were around 9 months old. Sweeps 2, 3 and 4 took place around ages 3, 5 and 7 years. The MCS provides a unique source of longitudinal measurements of SDQ at sweeps 2, 3 and 4. Longitudinal data on the SDQ are now available, providing a unique opportunity for studying the change in SDQ scores over time and how this is affected by risk factors and other family and child characteristics.

The data that we use in this paper are collected from children who participated in the first four sweeps of the MCS in England. Our study sample consists of 5000 MCS children in England, leading to a total sample size for the longitudinal data set equal to *n*=11 972 observations. The two outcomes of interest, emotional problems (measured by the SDQ internalizing score) and behavioural problems (measured by the SDQ externalizing score), were collected at ages 3, 5 and 7 years. The data consist of 3837 measurements at the first time point, 4314 at the second and 3821 at the third. Missing measurements are due to unit or item SDQ non‐response in a given time point, and previous MCS research demonstrates that less favourable family socio‐economic characteristics (e.g. lower parental qualifications) and ethnic minority backgrounds predict such non‐response (Flouri *et al*., 2014a, b). This is an additional reason for controlling for the effect of these covariates in the models that we present in Section 5 of the paper.

The key time varying explanatory variables are as follows. Adverse life events, *ALE 11*, were measured as the number (out of 11 events) of potentially stressful life events experienced by the family between two consecutive sweeps. The events, which were derived from available MCS data and based on the adverse life events scale of Tiet *et al*. (1998), were family member died, negative change in financial situation, new step‐parent, sibling left home, child got seriously sick or injured, divorce or separation, family moved, parent lost job, new natural sibling, new stepsibling and mother diagnosed with or treated for depression. Family poverty, measured by socio‐economic disadvantage, *SED 4*, combines information on overcrowding (more than 1.5 people per room excluding the bathroom and kitchen), not owning a home, receipt of means‐tested income support and income poverty (below the poverty line defined as 60% of the UK national median household income). Maternal depression, *kessm*, is measured by the Kessler score. An additional time varying variable is neighbourhood deprivation measured by the index of multiple‐deprivation score, *imdscore*. Furthermore, child's age (in years, centred near the mean age—*age year scal*) and the quadratic effect of child's age (*age2 year scal*) were included in the model. The time constant variables that we considered include maternal education (no qualification (baseline), university *degree* or General Certificate of Secondary Education, *GCSE*), ethnicity (non‐white (baseline) or *white*) and gender (female (baseline) or *male*). Finally, a design variable which allows for the stratification of the MCS sampling design was included in the model. The stratification variable of the MCS consists of three categories, namely the advantaged stratum (baseline category), the ethnic stratum, *Eng eth stratum*, and the disadvantaged stratum, *Eng dis stratum*.

Table 1 presents summary statistics for the two outcomes, SDQ internalizing and externalizing scores, and for some key continuous covariates. The asymmetry in the SDQ outcomes is noted by examining the mean–median relationship. The average of adverse life events, ALE 11, is 1.447 and the maximum is 7. The mean Kessler score, kessm, is 2.767 but there are cases with much higher scores of maternal depression with the maximum value equal to 24. 38% of children have mothers who hold a degree, 49% have mothers with General Certificate of Secondary Education or other qualification and 13% have mothers with no educational qualification. 51% of the children are males and 83% are of white background.

**Table 1:** Summary statistics for the MCS data

*Variable*	*Minimum*	*1st quartile*	*Median*	*Mean*	*3rd quartile*	*Maximum*
SDQ internalizing	0.00	1.00	2.00	2.690	4.00	18.00
SDQ externalizing	0.00	3.00	5.00	5.365	8.00	20.00
ALE 11	0.00	1.00	1.00	1.447	2.00	7.00
kessm	0.00	0.00	2.00	2.767	4.00	24.00
imdscore	1.00	3.00	5.00	5.222	8.00	10.00
SED 4	0.00	0.00	0.00	0.694	1.00	4.00

**Figure 2:**
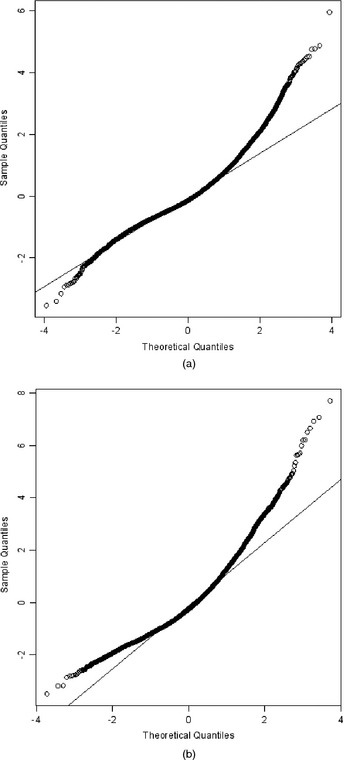
Normal probability plots of (a) level 1 and (b) level 2 residuals derived by fitting a two‐level linear mixed model for SDQ internalizing problems scores

**Figure 3:**
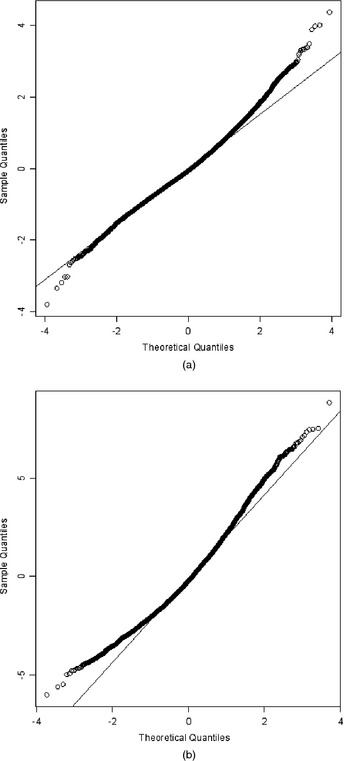
Normal probability plots of (a) level 1 and (b) level 2 residuals derived by fitting a two‐level linear mixed model for SDQ externalizing problems scores

The descriptive measures offer information about the unconditional distribution of the two SDQ outcomes. It is more appropriate, however, to study the conditional distribution of SDQ scores given a set of covariates. To do so we use a two‐level (level 1, measurement occasion; level 2, MCS child) random‐intercepts model for the two SDQ outcomes with random effects specified at the level of the MCS child. The model further adjusts for the effect of adverse life events, maternal depression, maternal education, socio‐economic disadvantage, neighbourhood deprivation, gender and ethnicity and accounts for the longitudinal structure of the data. Figs 2 and 3 present normal probability plots of level 1 and level 2 residuals. These indicate severe departures from the Gaussian assumptions of the random‐intercepts model for both SDQ outcomes. Hence, estimating a robust measure of central tendency and the quantiles of the conditional distribution of SDQ scores, given the covariates, is worth pursuing.

## 3 Multilevel models and robust estimation

Suppose that we have data on an outcome variable *y* and a set of covariates *x* for *n* individuals clustered within *d* groups. This can be either a longitudinal or a multilevel data set. A popular approach for modelling hierarchically structured data is to use a random‐effects model. In the simplest case we can define a random‐intercepts model

(1) $$y_{ij}=x_{ij}^{T}\beta +z_{ij}^{T}\gamma +\epsilon_{ij},i=1,\ldots ,n_{j},j=1,\ldots ,d,$$

where $x_{ij}$ is a *p*‐vector of **X**,***β*** is a *p*×1 vector of regression coefficients and $z_{ij}$ is a *d*×1 vector of group indicators used for defining the random part of the model. In addition, ***γ*** denotes a *d*×1 vector of group‐specific random effects, $\epsilon_{ij}$ is the individual random effect and we assume that $\gamma ∼N(0,\sigma_{\gamma }^{2}I_{d})$ and $\epsilon_{ij}∼N\left(0,\sigma_{\epsilon }^{2}\right)$. Here and throughout the paper $I_{k}$ is an identity matrix of size *k*. One way of obtaining estimates of the fixed effects and the variance components of model (1) is to employ maximum likelihood estimation. Assuming normality for the error components and *ε*⊥*γ*, the log‐likelihood function is

(2) $$l\left(\beta ,\sigma_{\gamma }^{2},\sigma_{\epsilon }^{2}\right)=-\frac{1}{2}\text{log}\left|V\right|-\frac{1}{2}{\left(y-X\beta \right)}^{T}V^{-1}\left(y-X\beta \right),$$

where **y** is the *n*×1 response vector, $V=\Sigma_{\epsilon }+Z\Sigma_{\gamma }Z^{T}$, $\Sigma_{\epsilon }=\sigma_{\epsilon }^{2}I_{n}$, $\Sigma_{\gamma }=\sigma_{\gamma }^{2}I_{d}$ and **Z** is an *n*×*d* matrix of known positive constants. Estimates of ***β***,***γ***, $\sigma_{\gamma }^{2}$ and $\sigma_{\epsilon }^{2}$ are obtained by differentiating the above log‐likelihood with respect to these parameters and then solving the estimating equations that are defined by setting these derivatives equal to 0. Predicted random effects are then obtained by using the maximum likelihood estimates of the fixed effects and the variance components. It is easy to see that in equation (2) we assume a squared loss function. In practice, however, data may contain outliers that invalidate the Gaussian assumptions. In such a case the estimated model parameters under equation (2) will be biased and inefficient (Richardson and Welsh, 1995). One approach to robust estimation of the random‐effects model that protects us against departures from normality is to use an alternative loss function in the log‐likelihood that grows along with the regression residuals at a slower rate than the squared loss function. This is the approach that was followed by Huggins (1993) and Richardson and Welsh (1995). In particular, robust maximum likelihood estimation for the random‐effects model is performed by maximizing the modified log‐likelihood function

(3) $$l\left(\beta ,\sigma_{\gamma }^{2},\sigma_{\epsilon }^{2}\right)=-\frac{K_{1}}{2}\text{log}\left|V\right|-\rho \left(r\right),$$

where $r=V^{-1/2}\left(y-X\beta \right)$, *ρ* is a loss function, *ψ* is its derivative and $K_{1}=E\left[\varepsilon \psi {\left(\varepsilon \right)}^{T}\right]$ with $\varepsilon ∼N(0,{\;I}_{n})$. Robust estimates of ***β***, $\sigma_{\gamma }^{2}$ and $\sigma_{\epsilon }^{2}$ are obtained by solving the estimating equations defined by setting the derivatives of the modified log‐likelihood with respect to the parameters equal to 0. This is the robust maximum likelihood proposal I by Richardson and Welsh (1995). To ensure robustness, *ψ*(**r**) and $r^{T}\psi \left(r\right)$ must be bounded. A bounded *ρ*‐function leading to a redescending *ψ* fulfils these conditions. An alternative to the use of a redescending *ψ*‐function is to solve the following estimating equations for $\sigma_{\gamma }^{2}$ and $\sigma_{\epsilon }^{2}$:

(4) $$\frac{1}{2}\psi {\left(r\right)}^{T}V^{-1/2}ZZ^{T}V^{-1/2}\psi \left(r\right)-\frac{K_{2}}{2}\text{tr}\left(V^{-1}ZZ^{T}\right)$$

where $K_{2}=E\left[\psi \left(\varepsilon \right)\psi {\left(\varepsilon \right)}^{T}\right]$ with $\varepsilon ∼N(0,I_{n})$. Richardson and Welsh (1995) called this robust maximum likelihood proposal II. It can be viewed as a generalization of Huber's proposal II (Huber, 1981). However, there is no likelihood function that has expression (4) as its derivative with respect to the variance components. For details see Richardson and Welsh (1995).

Robust predicted random effects can be obtained by solving for ***γ*** in the estimating equation (Fellner, 1986)

$$Z^{T}\Sigma_{\epsilon }^{-1/2}\psi \left\{\Sigma_{\epsilon }^{-1/2}\left(y-X\beta -Z\gamma \right)\right\}-\Sigma_{\gamma }^{-1/2}\psi \left(\Sigma_{\gamma }^{-1/2}\gamma \right)=0.$$

An alternative robust estimation approach that can potentially lead to more efficient estimates of the variance components when there is a small number of groups or when groups contain a small number of observations is the robust restricted maximum likelihood approach (Richardson and Welsh, 1995; Staudenmayer *et al*., 2009).

## 4 *M*‐quantile regression and extensions to ***M***‐quantile random‐effects regression

In this paper we are interested in describing the relationship between *y* and *x* not only at the centre of the conditional distribution of *y* given *x* but also at other parts of this distribution. In this section we start by reviewing *M*‐quantile regression and subsequently extend this to *M*‐quantile random‐effects regression.

### 4.1 M‐quantile regression

The classic regression model summarizes the behaviour of the mean of a random variable *y* at each point in a set of covariates *x*. This provides a rather incomplete picture, in much the same way as the mean may offer an incomplete picture of a distribution. Instead, quantile regression summarizes the behaviour at different parts (e.g. quantiles) of the distribution of *y* at each point in the set of the *x*s.

In the linear case, quantile regression leads to a family of hyperplanes indexed by a real number *q* ∈ (0,1). For a given value of *q*, the corresponding model shows how the *q*th quantile of the conditional distribution of *y* varies with *x*. For example, if *q*=0.5 the quantile regression hyperplane shows how the median of the conditional distribution changes with *x*. Similarly, for *q*=0.1 the quantile regression hyperplane separates the lower 10% of the conditional distribution from the remaining 90%.

As we mentioned in Section 1, quantile regression can be viewed as a generalization of median regression. In the same way, expectile regression (Newey and Powell, 1987) is a ‘quantile‐like’ generalization of mean regression. MQ regression (Breckling and Chambers, 1988) integrates these concepts within a framework defined by a quantile‐like generalization of regression based on influence functions (*M*‐regression). The MQ of order *q* of the conditional density of *y* given the set of covariates *x*,* f*(*y*|*x*), is defined as the solution ${\text{MQ}}_{y}\left(q|x;\psi \right)$ of an estimating equation $\int \psi_{q}\left\{y-{\text{MQ}}_{y}\left(q|x;\psi \right)\right\}f\left(y|x\right)dy=0$, where $\psi_{q}$ denotes an asymmetric influence function, which is the derivative of an asymmetric loss function $\rho_{q}$. In particular, suppose that $(x_{i}^{T},y_{i}),i=1,\ldots ,n$, indexes the units of a random sample consisting of *n* observations from the target population, $x_{i}^{T}$ are row *p*‐vectors of a known design matrix **X** and $y_{i}$ is a scalar response variable corresponding to a realization of a continuous random variable with unknown continuous cumulative distribution function *F*; a linear MQ regression model for $y_{i}$ given $x_{i}$ is one where we assume that

(5) $${\text{MQ}}_{y_{i}}\left(q|x_{i};\psi \right)=x_{i}^{T}\beta_{\psi q},$$

i.e. we allow a different set of *p* regression parameters for each value of *q* ∈ (0,1). Estimates of $\beta_{\psi q}$ are obtained by minimizing

(6) $$\sum_{i=1}^{n}\rho_{q}\left(y_{i}-x_{i}^{T}\beta_{\psi q}\right).$$

Different regression models can be defined as special cases of expression (6). In particular, by varying the specifications of the asymmetric loss function $\rho_{q}$ we obtain the expectile, MQ and quantile regression models as special cases. When $\rho_{q}$ is the squared loss function we obtain the linear expectile regression model if *q*≠0.5 (Newey and Powell, 1987) and the standard linear regression model if *q*=0.5. When $\rho_{q}$ is the loss function that was described by Koenker and Bassett (1978) we obtain linear quantile regression. Throughout this paper we shall take the linear MQ regression model to be defined by equation (5) when $\rho_{q}$ is the Huber loss function (Breckling and Chambers, 1988):

$$\rho_{q}\left(u\right)=\left\{\begin{array}{cc} (2c|u|-c^{2})\left\{qI\left(u>0\right)+\left(1-q\right)I\left(u⩽0\right)\right\} & |u|>c,\\ u^{2}\left\{qI\left(u>0\right)+\left(1-q\right)I\left(u⩽0\right)\right\} & |u|⩽c, \end{array}\right.$$

where *c* is the tuning constant, which is bounded away from zero. Setting the first derivative of expression (6) equal to 0 leads to the estimating equations

(7) $$\sum_{i=1}^{n}\psi_{q}\left(r_{iq}\right)x_{i}=0,$$

where $r_{iq}=y_{i}-x_{i}^{T}\beta_{\psi q}$, $\psi_{q}\left(r_{iq}\right)=2\psi \left(s^{-1}r_{iq}\right)\left\{qI\left(r_{iq}>0\right)+\left(1-q\right)I\left(r_{iq}⩽0\right)\right\}$ and *ψ*(*u*)=*u* *I*(−*c*⩽*u*⩽*c*)+*c* sgn(*u*) *I*(|*u*|>*c*) and *s*>0 is a suitable estimate of scale. For example, in the case of robust regression, $s=\text{median}|r_{iq}|/0.6745$. Provided that the tuning constant *c* is strictly greater than 0, estimates of $\beta_{\psi q}$ are obtained by using iterative weighted least squares. The estimation algorithm is implemented in R by a simple modification of the iterative weighted least squares algorithm used for fitting standard *M*‐regression by using the function rlm (Venables and Ripley (2002), section 8.3). Note that this guarantees a unique solution (Kokic *et al*., 1997) when a continuous monotone influence function (e.g. Huber proposal 2 with *c*>0) is used. The tuning constant *c* can be used to trade robustness for efficiency in the MQ regression fit, with increasing robustness and decreasing efficiency as we move towards quantile regression (*c* chosen to be positive and close to 0) and decreasing robustness and increasing efficiency as we move towards expectile regression (*c* chosen to be large and positive). Since we cannot set *c* to 0, it is not possible to use iterative weighted least squares for quantile regression. However, by setting *c* to be very large and positive we define an expectile regression model. The flexibility of MQ regression is of particular importance for the present paper as this will also allow us to define an expectile random‐effects regression model.

### 4.2 M‐quantile random‐effects regression

In this section we assume that the data have group structure (multilevel or longitudinal) as in Section 3, which is of substantive interest. We extend the linear specification (5) to allow for the inclusion of random effects when modelling the MQs of the target distribution. This extension can be useful when analysing multilevel‐type data in which case the random effects aim at capturing unobserved heterogeneity. In the simplest case one can include a group‐specific random intercept in the linear specification for the MQs (5) as follows:

(8) $${\text{MQ}}_{y_{ij}}\left(q|x_{ij},\gamma ,j;\psi \right)=x_{ij}^{T}\beta_{\psi q}+z_{ij}^{T}\gamma ,$$

where ***γ*** is a *d*×1 vector of group random effects.

For fitting equation (8) we propose the use of estimating equations based on asymmetric loss functions. This novel approach for modelling location parameters is what we call MQRE regression. Unlike Geraci and Bottai (2007, 2014) who utilized the link between maximum likelihood estimation under the asymmetric Laplace distribution and quantile regression, we remain within the *M*‐estimation framework. Since the estimating equations that are obtained from the modified log‐likelihood function (3) are susceptible to multiple solutions, we start from the robust maximum likelihood proposal II and following Sinha and Rao (2009) we note that one can extend the idea of asymmetric weighting of residuals by defining the following modified estimating equations for estimating the regression coefficients and the variance parameters:

(9) $$X^{T}V_{q}^{-1}U_{q}^{1/2}\psi_{q}\left(r_{q}\right)=0,$$

(10) $$\begin{array}{cc} \frac{1}{2}\psi_{q}{\left(r_{q}\right)}^{T}U_{q}^{1/2}V_{q}^{-1}ZZ^{T}V_{q}^{-1}U_{q}^{1/2}\psi_{q}\left(r_{q}\right)-\frac{K_{2q}}{2}\text{tr}\left(V_{q}^{-1}ZZ^{T}\right) & =0,\\ \frac{1}{2}\psi_{q}{\left(r_{q}\right)}^{T}U_{q}^{1/2}V_{q}^{-1}V_{q}^{-1}U_{q}^{1/2}\psi_{q}\left(r_{q}\right)-\frac{K_{2q}}{2}\text{tr}\left(V_{q}^{-1}\right)=0, \end{array}$$

where $r_{q}=U_{q}^{-1/2}\left(y-X\beta_{\psi q}\right)$ is a vector of scaled residuals with components $r_{ijq}$, $U_{q}$ is a diagonal matrix with diagonal elements $u_{ijq}$ equal to the diagonal elements of the covariance matrix $V_{q}$ and $\beta_{\psi q}$ is the *p*×1 vector of MQ regression coefficients. Here $V_{q}=\Sigma_{\epsilon_{q}}+Z\Sigma_{\gamma_{q}}Z^{T}$, $\Sigma_{\gamma_{q}}=\sigma_{\gamma_{q}}^{2}I_{d}$, $\Sigma_{\epsilon_{q}}=\sigma_{\epsilon_{q}}^{2}I_{n}$ and $\sigma_{\epsilon_{q}}^{2}$ and $\sigma_{\gamma_{q}}^{2}$ are the MQ‐specific variance parameters. Finally, the component $K_{2q}=E\left[\psi_{q}\left(\varepsilon \right)\psi_{q}{\left(\varepsilon \right)}^{T}\right]$ with $\varepsilon ∼N(0,I_{n})$.

For solving equations (9) and (10) we adopt a Newton–Raphson algorithm and the fixed point iterative method (Anderson, 1973). In particular, the fixed effects are estimated by using a Newton–Raphson algorithm whereas the variance parameters are estimated by using a fixed point algorithm. Using the Newton–Raphson optimization method for estimating the variance parameters can cause convergence problems. It is therefore preferable to use a technique that is derivative free such as the fixed point iterative method. The steps of the estimation algorithm are described in Appendix A.

Although prediction of the random‐effects vector *γ* in equation (8) is not the primary focus in this paper, we outline a possible solution. In particular, the simplest solution is to predict the random effects by using a modified Fellner equation (Fellner, 1986) at each value of *q*. The issue of predicting the random effects is also discussed in Geraci and Bottai (2014) and our proposed solution is similar to the solution that they proposed. However, the approach that we use for fitting equation (8) separately at each *q* makes *γ* depend on *q* since these are functions of MQ‐specific parameters (see equation (12) in Appendix A). This raises the question of how one then combines these different *q*‐specific estimates of *γ* since they will clearly be correlated over *q*. An alternative approach that avoids this issue would be to modify the *q*‐specific fitting process that was described above to allow for a common *γ* across *q* as in equation (8) or by imposing a suitable group‐specific ordering over these *q*‐specific predicted values. Both approaches can be potentially achieved by adding suitable constraints to this fitting procedure. Exploring alternative approaches to the prediction of the random‐effects vector *γ* in equation (8) remains an open problem that we are currently investigating.

The estimating equations under expression (2) can be obtained as a special case of equations (9) and (10) for specific choices of $\rho_{q}$ and *q*. In particular, when *q*=0.5 and $\rho_{q}$ is the squared loss function we obtain the estimating equations under expression (2). When *q*=0.5 and we use a loss function other than the squared loss, e.g. the Huber loss function, we obtain the estimating equations of the robust maximum likelihood proposal II. For *q*‐values other than 0.5 and for different choices of *ρ*, solving equations (9) and (10) will provide estimates of fixed effects $\beta_{\psi q}$ and variance parameters, $\sigma_{\epsilon_{q}}^{2}$ and $\sigma_{\gamma_{q}}^{2}$, respectively. More specifically, using a squared loss function in equations (9) and (10) at *q*≠0.5 results in the expectile random‐effects regression whereas using the Huber loss function in equations (9) and (10) results in MQRE regression.

Inference for the parameters of the linear random‐effects model when the Gaussian assumptions hold has been studied by Hartley and Rao (1967) and Miller (1977). Huber (1967) showed the consistency and asymptotic normality of ‘maximum‐likelihood‐type’ estimators (*M*‐estimators). The work of Huber (1967) is specifically linked to robust estimation problems and his arguments were used by Welsh and Richardson (1997) to propose robust inference for the parameters of the linear random‐effects model. Inference for the parameters of the MQ and expectile random‐effects regression is based on a Taylor series approximation. Details are given in Appendix B. An alternative approach for inference is by using the bootstrap. A recent example of the use of the parametric bootstrap in the case of robust estimation of the parameters of a random‐effects model has been given by Sinha and Rao (2009). A significant drawback to using the bootstrap in the case of MQRE regression is the computation time that is required for performing a large number of bootstrap replications. We have performed some limited empirical assessment of the parametric bootstrap procedure and the results are consistent with those obtained from the analytic approximation based on the Taylor series expansion.

## 5 Analysis of the Millennium Cohort Study data

In this section we present longitudinal modelling of the SDQ internalizing and externalizing scores for our sample of MCS children. In light of the substantive literature that was described in Section 1, we are mainly interested in the effect of family and neighbourhood risk factors on young children's emotional and behavioural problems. We modelled the effects of the following time varying and time constant variables on SDQ scores at ages 3, 5 and 7 years: adverse life events, socio‐economic disadvantage, maternal depression, maternal education, ethnicity, gender, neighbourhood deprivation, child's age and the quadratic effect of child's age. Details about these variables are provided in Section 2. We further control for the effects of stratification by including the stratification variable in the models.

For longitudinal modelling of location parameters of the distributions of the internalizing and externalizing scores we used the following models:

- the proposed MQRE model (Section 4) with random intercepts specified at the level of the MCS member,
- the LQMM (Geraci and Bottai, 2014) with random intercepts specified at the level of the MCS member,
- the LQMM (Geraci and Bottai, 2014) with random intercepts specified at the level of the MCS member and random slopes (coefficients) specified for age and
- the linear random effects (LRE) model (1) for the mean (produced by using the lme function in R).

In addition to the MQRE model proposed, the reason for using the LQMM (Geraci and Bottai, 2014) in the application is to inform the prospective data analyst about the range of modelling tools that are available.

The lqmm function in R, that estimates the LQMM (Geraci and Bottai, 2014), allows for the specification of both random intercepts and random coefficients. In contrast, MQRE regression allows only for random intercepts. Random intercepts imply a uniform (exchangeable) correlation structure whereas random slopes allow the correlation structure to depend on age, which may offer a more realistic structure for repeated measures data. Although possible, allowing for random slopes in quantile random‐effects models is complex and can potentially result in convergence problems when fitting the model. In contrast, quantile models with a random‐intercepts specification have a correlation structure that is simpler to estimate while allowing for modelling the entire conditional distribution of the outcome. The MQRE and LQMM results are not directly comparable as these models are targeting different location parameters. However, both models attempt to model location parameters that are associated with the same part of the conditional distribution of SDQ scores.

### 5.1 Results for externalizing scores

Table 2 presents the results of the MQRE random‐intercepts model. The parameters of the MQRE model are estimated by using the algorithm in Appendix A (see also Section 2). The intercepts are estimates of location parameters (MQs in the case of MQRE regression) of the SDQ externalizing score of a child at age 5 years (age is centred) when setting the categorical covariates to the corresponding baseline values and the continuous variables to 0. The estimated regression coefficients are consistent with what theory predicts. Males appear to have higher SDQ scores compared with females. Increasing adverse life events, socio‐economic disadvantage, maternal depression, neighbourhood deprivation and lower maternal education are associated with higher SDQ scores. In addition, after controlling for neighbourhood and family characteristics, we do not find an ethnicity effect.

**Table 2:** Results—MQRE random‐intercepts model for externalizing scores^a^

*Variable*	*Results for the following values of q*:									
	*0.1*		*0.25*		*0.5*		*0.75*		*0.9*	
Intercept	1.972	(0.197)	2.959	(0.208)	4.265	(0.231)	5.774	(0.276)	7.174	(0.341)
age year scal	−0.349	(0.013)	−0.401	(0.013)	−0.456	(0.015)	−0.476	(0.018)	−0.455	(0.023)
age2 year scal	0.136	(0.009)	0.171	(0.009)	0.219	(0.009)	0.258	(0.011)	0.274	(0.016)
ALE 11	0.074	(0.025)	0.098	(0.025)	0.131	(0.027)	0.172	(0.033)	0.208	(0.043)
SED 4	0.089	(0.037)	0.120	(0.038)	0.180	(0.041)	0.250	(0.048)	0.301	(0.057)
kessm	0.150	(0.011)	0.180	(0.011)	0.211	(0.012)	0.236	(0.015)	0.265	(0.019)
degree	−1.063	(0.133)	−1.430	(0.143)	−1.875	(0.160)	−2.180	(0.185)	−2.298	(0.217)
GCSE	−0.421	(0.130)	−0.632	(0.140)	−0.917	(0.154)	−1.078	(0.174)	−1.110	(0.198)
white	0.024	(0.113)	0.061	(0.119)	0.126	(0.135)	0.185	(0.161)	0.179	(0.201)
male	0.658	(0.065)	0.804	(0.071)	0.968	(0.081)	1.094	(0.099)	1.191	(0.121)
imdscore	−0.022	(0.014)	−0.026	(0.015)	−0.035	(0.017)	−0.050	(0.021)	−0.049	(0.026)
Eng eth stratum	0.110	(0.140)	0.212	(0.149)	0.300	(0.168)	0.242	(0.197)	0.139	(0.243)
Eng dis stratum	0.160	(0.083)	0.267	(0.092)	0.401	(0.105)	0.486	(0.131)	0.579	(0.160)
$\sigma_{\gamma_{q}}^{2}$	0.708	—	2.564	—	5.718	—	4.754	—	2.127	—
$\sigma_{\epsilon_{q}}^{2}$	0.975	—	2.633	—	4.762	—	4.073	—	2.388	—

a Point estimates with standard errors in parentheses.

**Figure 4:**
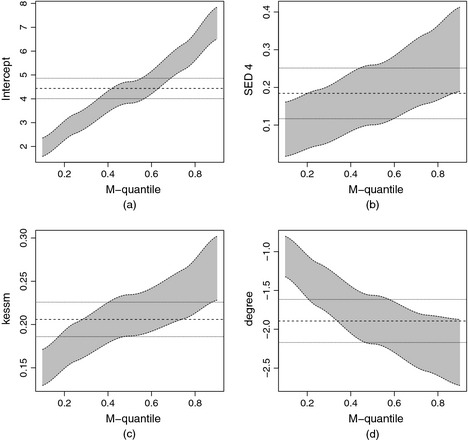
Confidence intervals for (a) the intercept, (b) SED 4, (c) kessm and (d) degree

The grey area in Fig. 4 displays 95% confidence intervals of the MQRE parameters for various MQs for some selected risk factors (maternal depression, socio‐economic disadvantage and higher *versus* no maternal educational qualifications). The MQRE standard errors are computed by using the methodology in Section 4.2 and in Appendix B. The bold dotted curve presents the corresponding parameter estimates that were obtained under the LRE model. This model is targeting the conditional expectation of the externalizing score given the explanatory variables. The two dotted curves around the bold dotted curve present the upper and lower bounds of 95% confidence intervals. The most interesting aspect of this analysis is that it allows for the estimation of the effect of covariates at different parts of the distribution of SDQ scores. As expected, increasing values for the risk factors such as SED 4, maternal depression and lower maternal education are associated with increased SDQ scores. The effect of these covariates appears to be more pronounced when looking at the upper tail compared with the lower tail of the distribution. For example, the disparity in the externalizing scores of children with mothers who have higher educational qualifications, compared with children with mothers who have no educational qualifications, is smaller at the lower part of the distribution compared with the upper part of the distribution. This may suggest that the protective role of higher maternal education is more pronounced for children with more externalizing problems. Maternal depression also appears to have a stronger effect at the top end, compared with the lower end, of the distribution. We revisit these results in the next section and we draw some comparisons between the externalizing scores results and the results for the internalizing scores by using relevant substantive literature.

The results for the LQMM are shown in Table 3 for the random‐intercepts model and in Table 4 for the random‐slopes model. Standard errors in this case are computed by using the bootstrap estimator that was proposed by Geraci and Bottai (2014). To start we note the differences between the parameter estimates of the median and mean models. The estimated level 2 variance component from the median model is smaller than the corresponding estimate from the mean model (see $\sigma_{\gamma_{q}}^{2}$ in Table 3). The MQRE level 2 variance component lies between the LQMM and the LRE components (see $\sigma_{\gamma_{q}}^{2}$ in Table 2). These results are as expected and the differences may be explained by the diagnostics that we presented in Section 2. As expected, in most cases the standard errors of the robust estimates are also somewhat higher than the corresponding standard error estimates from the LRE model.

**Table 3:** Results—LQMM random‐intercepts model for externalizing scores^a^

*Variable*	*Results for the following values of q*:					
	*0.1*	*0.25*	*0.5*	*LRE—mean*	*0.75*	*0.9*
Intercept	1.905	3.174	4.065	4.434	5.033	6.073
	(0.298)	(0.269)	(0.274)	(0.218)	(0.282)	(0.328)
age year scal	−0.336	−0.374	−0.433	−0.442	−0.467	−0.478
	(0.026)	(0.019)	(0.020)	(0.019)	(0.019)	(0.033)
age2 year scal	0.141	0.162	0.212	0.222	0.255	0.287
	(0.017)	(0.010)	(0.011)	(0.010)	(0.015)	(0.018)
ALE 11	0.121	0.128	0.127	0.134	0.187	0.124
	(0.051)	(0.028)	(0.043)	(0.025)	(0.042)	(0.046)
SED 4	0.085	0.132	0.252	0.184	0.260	0.294
	(0.060)	(0.059)	(0.049)	(0.034)	(0.096)	(0.069)
kessm	0.169	0.208	0.215	0.206	0.252	0.233
	(0.026)	(0.019)	(0.016)	(0.010)	(0.017)	(0.023)
degree	−1.212	−1.384	−1.660	−1.893	−1.546	−1.828
	(0.176)	(0.209)	(0.180)	(0.141)	(0.161)	(0.191)
GCSE	−0.351	−0.862	−0.829	−0.905	−0.570	−0.743
	(0.178)	(0.166)	(0.161)	(0.129)	(0.167)	(0.180)
white	0.119	0.057	0.283	0.142	0.311	0.629
	(0.146)	(0.138)	(0.164)	(0.134)	(0.135)	(0.163)
male	0.653	0.756	0.958	0.980	1.189	1.122
	(0.139)	(0.097)	(0.077)	(0.082)	(0.119)	(0.124)
imdscore	−0.006	−0.029	−0.037	−0.036	−0.048	−0.035
	(0.030)	(0.026)	(0.018)	(0.017)	(0.031)	(0.043)
Eng eth stratum	−0.069	−0.047	0.296	0.271	0.543	0.625
	(0.159)	(0.209)	(0.168)	(0.167)	(0.166)	(0.164)
Eng dis stratum	−0.017	0.282	0.282	0.422	0.827	0.903
	(0.111)	(0.106)	(0.103)	(0.106)	(0.114)	(0.111)
$\sigma_{\gamma_{q}}^{2}$	3.369	4.392	5.199	6.164	6.163	6.717
	—	—	—	—	—	—

a Point estimates with standard errors in parentheses.

**Table 4:** Results—LQMM random‐slopes model for externalizing scores^a^

*Variable*	*Results for the following values of q*:					
	*0.1*	*0.25*	*0.5*	*LRE—mean*	*0.75*	*0.9*
Intercept	1.937	3.200	4.057	4.454	5.035	5.906
	(0.309)	(0.264)	(0.298)	(0.217)	(0.280)	(0.310)
age year scal	−0.353	−0.396	−0.432	−0.442	−0.467	−0.471
	(0.030)	(0.018)	(0.019)	(0.014)	(0.023)	(0.032)
age2 year scal	0.184	0.194	0.211	0.225	0.255	0.215
	(0.027)	(0.013)	(0.011)	(0.009)	(0.022)	(0.035)
ALE 11	0.122	0.115	0.132	0.130	0.187	0.134
	(0.055)	(0.030)	(0.041)	(0.025)	(0.042)	(0.050)
SED 4	0.064	0.116	0.258	0.173	0.260	0.308
	(0.064)	(0.060)	(0.059)	(0.034)	(0.101)	(0.093)
kessm	0.202	0.195	0.214	0.201	0.252	0.241
	(0.025)	(0.018)	(0.014)	(0.010)	(0.020)	(0.028)
degree	−1.244	−1.446	−1.665	−1.902	−1.546	−1.805
	(0.181)	(0.206)	(0.187)	(0.141)	(0.168)	(0.190)
GCSE	−0.352	−0.774	−0.857	−0.919	−0.570	−0.741
	(0.182)	(0.167)	(0.176)	(0.129)	(0.164)	(0.194)
white	0.200	0.130	0.299	0.149	0.309	0.459
	(0.150)	(0.143)	(0.186)	(0.134)	(0.142)	(0.155)
male	0.670	0.764	0.964	0.993	1.189	1.169
	(0.140)	(0.096)	(0.086)	(0.082)	(0.136)	(0.123)
imdscore	−0.005	−0.028	−0.040	−0.036	−0.048	0.001
	(0.028)	(0.025)	(0.018)	(0.017)	(0.029)	(0.045)
Eng eth stratum	−0.071	0.062	0.305	0.274	0.541	0.683
	(0.162)	(0.215)	(0.174)	(0.167)	(0.161)	(0.185)
Eng dis stratum	0.111	0.233	0.289	0.422	0.826	0.830
	(0.115)	(0.100)	(0.110)	(0.106)	(0.121)	(0.113)
$\sigma_{\gamma_{q}}^{2}$	3.996	4.631	5.139	6.478	6.163	6.960
	—	—	—	—	—	—
$\sigma_{\gamma_{q}}^{2}$ age	0.158	0.179	0.000	0.263	0.000	0.000
	—	—	—	—	—	—

a Point estimates with standard errors in parentheses.

The estimated intercepts from the LQMM random‐intercepts model (Table 3) present estimates of the corresponding quantiles of the SDQ externalizing scores distribution for a baseline MCS child. The asymmetry in this distribution is reflected by the mean–median relationship, which is consistent with the findings in Section 2. The results of the LQMM random‐intercepts model are comparable with the results obtained with MQRE regression. As we discussed earlier, the LQMM and MQRE regression target different population location parameters. The LQMM results are easier to interpret. The MQRE model, in contrast, targets the same part of the conditional distribution as the LQMM but the interpretation of the results is less intuitive. From the perspective of the data analyst, however, both models can be used to look at the entire distribution of the outcome accounting for the longitudinal structure of the data. Currently, one methodological advantage of the LQMM approach is that it allows for the specification of more complex correlation structures (e.g. random slopes). Table 4 presents the results from the LQMM that, in addition to a random intercept, includes a random slope on age. However, when fitting the random‐slopes model we experienced slow convergence for quantiles at the tails of the distribution whereas for the median fit the estimated variance component of the random slope is very close to 0. These results suggest that a random‐intercepts specification may present a more feasible approach that allows at the same time for modelling different parts of the distribution of externalizing scores.

### 5.2 Results for internalizing scores

**Figure 5:**
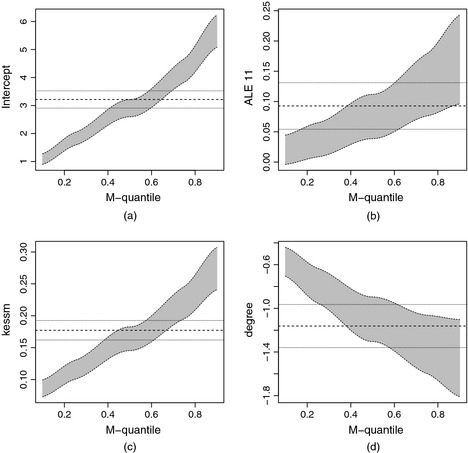
Confidence intervals for (a) the intercept, (b) ALE 11, (c) kessm and (d) degree

Table 5 presents the results of the random‐intercepts MQRE. As with the externalizing problems, the estimated regression coefficients for internalizing scores are consistent with child development theory. The grey area in Fig. 5 displays 95% confidence intervals of the MQRE parameters for various quantiles and for selected key risk factors.

**Table 5:** Results—MQRE random‐intercepts model for internalizing scores^a^

*Variable*	*Results for the following values of q*:									
	*0.1*		*0.25*		*0.5*		*0.75*		*0.9*	
Intercept	1.088	(0.097)	1.813	(0.116)	2.904	(0.157)	4.249	(0.214)	5.656	(0.293)
age year scal	−0.042	(0.007)	−0.049	(0.008)	−0.043	(0.010)	0.005	(0.014)	0.089	(0.022)
age2 year scal	0.034	(0.005)	0.050	(0.005)	0.075	(0.007)	0.099	(0.009)	0.113	(0.015)
ALE 11	0.020	(0.012)	0.037	(0.014)	0.075	(0.019)	0.128	(0.026)	0.169	(0.037)
SED 4	0.026	(0.018)	0.035	(0.021)	0.061	(0.027)	0.106	(0.037)	0.093	(0.051)
kessm	0.086	(0.007)	0.116	(0.008)	0.164	(0.009)	0.221	(0.013)	0.274	(0.017)
degree	−0.573	(0.068)	−0.794	(0.081)	−1.100	(0.104)	−1.330	(0.136)	−1.456	(0.180)
GCSE	−0.372	(0.065)	−0.531	(0.078)	−0.736	(0.101)	−0.834	(0.131)	−0.813	(0.169)
white	−0.232	(0.058)	−0.327	(0.070)	−0.484	(0.096)	−0.664	(0.132)	−0.805	(0.176)
male	0.062	(0.031)	0.085	(0.038)	0.136	(0.051)	0.190	(0.071)	0.271	(0.100)
imdscore	−0.019	(0.007)	−0.027	(0.008)	−0.047	(0.011)	−0.076	(0.015)	−0.106	(0.023)
Eng eth stratum	0.134	(0.072)	0.204	(0.087)	0.303	(0.115)	0.271	(0.160)	0.159	(0.216)
Eng dis stratum	0.096	(0.039)	0.124	(0.048)	0.149	(0.065)	0.121	(0.094)	0.111	(0.134)
$\sigma_{\gamma_{q}}^{2}$	0.147	—	0.631	—	1.958	—	2.108	—	1.074	—
$\sigma_{\epsilon_{q}}^{2}$	0.285	—	0.989	—	2.480	—	2.723	—	1.895	—

a Point estimates with standard errors in parentheses.

After controlling for family and area characteristics, socio‐economic disadvantage is significantly associated with internalizing scores only at *q*=0.5 and *q*=0.75. This is in contrast with the more pronounced effect of socio‐economic disadvantage, across the distribution, that we found for externalizing scores. This is in line with findings from a large number of studies showing that, in general, poverty and material deprivation are less consistently and less strongly associated with children's internalizing problems compared with externalizing problems (Costello *et al*., 2010; Dearing *et al*., 2006). This differential effect of poverty is not surprising considering that, in general, genetic influences are modest and environmental influences are large for antisocial behaviour relative to other childhood disorders (Plomin *et al*., 1997). Externalizing problems may therefore be more malleable than internalizing problems in response to environmental changes.

In contrast, maternal depression is significantly associated with increased internalizing scores. This effect is also clearly more pronounced (compared with the results for externalizing scores) at the top end of the distribution. Although maternal depression is related to both externalizing and internalizing problems, depression as a construct is more associated with internalizing problems such as emotional symptoms. The top end of the distribution may represent children with abnormal problems (or disorders) whose scores are likely to be affected by several factors contributing to maternal depression effects including genetic transmission or poor parenting (Goodman and Gotlib, 1999). Importantly, there is recent evidence that the mechanisms of risk from parental depression to externalizing problems are different from those for internalizing problems. For example, Silberg *et al*. (2010) showed that, whereas internalizing problems were accounted for solely by environmental factors, both environmental and genetic factors were significant in the association between parental depression and childhood externalizing problems. As with externalizing problems, the protective effect of higher maternal education is present also for internalizing problems.

Turning now to the results from the LQMM random‐intercepts model in Table 6, again we note that these are in line with the MQRE results. However, with the LQMM we experienced more problems with the convergence of the algorithm, which demonstrates that estimating more extreme quantiles can be sometimes challenging. In contrast, estimation with MQRE regression was smoother but this comes at the cost of modelling location parameters that are more difficult to interpret. Table 7 presents the results from the LQMM that includes a random slope on age. For this model we experienced slower convergence when estimating the lower quantiles. Finally, the comments on the relationship between the mean and median fits in Section 5.1 also apply to the results for the internalizing scores.

**Table 6:** Results—LQMM random‐intercepts model for internalizing scores^a^

*Variable*	*Results for the following values of q*:					
	*0.1*	*0.25*	*0.5*	*LRE—mean*	*0.75*	*0.9*
Intercept	1.746	1.680	2.934	3.215	3.870	4.355
	(0.130)	(0.261)	(0.161)	(0.157)	(0.220)	(0.184)
age year scal	−0.000	−0.000	−0.037	−0.008	−0.041	−0.063
	(0.000)	(0.009)	(0.012)	(0.010)	(0.016)	(0.023)
age2 year scal	0.000	0.000	0.052	0.083	0.096	0.125
	(0.000)	(0.008)	(0.011)	(0.008)	(0.010)	(0.015)
ALE 11	0.000	0.000	0.048	0.093	0.104	0.138
	(0.000)	(0.010)	(0.021)	(0.020)	(0.035)	(0.034)
SED 4	−0.000	0.000	0.099	0.061	0.071	0.105
	(0.000)	(0.016)	(0.039)	(0.026)	(0.052)	(0.051)
kessm	0.000	0.000	0.157	0.177	0.211	0.234
	(0.000)	(0.034)	(0.012)	(0.008)	(0.016)	(0.021)
degree	−1.012	−0.687	−0.897	−1.162	−1.142	−0.978
	(0.061)	(0.114)	(0.134)	(0.101)	(0.142)	(0.121)
GCSE	−1.012	−0.687	−0.702	−0.718	−0.702	−0.348
	(0.061)	(0.127)	(0.116)	(0.092)	(0.162)	(0.124)
white	−0.735	0.008	−0.452	−0.559	−0.499	−0.220
	(0.134)	(0.204)	(0.108)	(0.095)	(0.142)	(0.124)
male	0.000	0.000	0.101	0.173	0.153	0.307
	(0.000)	(0.009)	(0.059)	(0.058)	(0.079)	(0.099)
imdscore	0.000	0.000	−0.030	−0.059	−0.032	−0.095
	(0.000)	(0.007)	(0.014)	(0.012)	(0.019)	(0.017)
Eng eth stratum	−0.276	0.008	0.217	0.273	0.592	0.786
	(0.115)	(0.074)	(0.110)	(0.119)	(0.148)	(0.115)
Eng dis stratum	0.000	0.000	0.058	0.148	0.163	0.363
	(0.000)	(0.016)	(0.065)	(0.075)	(0.097)	(0.119)
$\sigma_{\gamma_{q}}^{2}$	0.000	0.750	2.280	2.722	3.562	4.240
	—	—	—	—	—	—

a Point estimates with standard errors in parentheses.

**Table 7:** Results—LQMM random‐slopes model for internalizing scores^a^

*Variable*	*Results for the following values of q*:					
	*0.1*	*0.25*	*0.5*	*LRE—mean*	*0.75*	*0.9*
Intercept	1.749	1.671	2.942	3.271	4.036	4.311
	(0.129)	(0.175)	(0.152)	(0.156)	(0.232)	(0.256)
age year scal	−0.000	−0.000	−0.036	−0.006	−0.018	−0.020
	(0.000)	(0.010)	(0.012)	(0.011)	(0.018)	(0.025)
age2 year scal	−0.000	0.000	0.052	0.085	0.095	0.073
	(0.000)	(0.008)	(0.011)	(0.007)	(0.011)	(0.022)
ALE 11	−0.000	0.000	0.047	0.085	0.086	0.112
	(0.000)	(0.009)	(0.023)	(0.019)	(0.035)	(0.043)
SED 4	−0.000	0.000	0.100	0.055	0.067	0.141
	(0.000)	(0.017)	(0.037)	(0.026)	(0.048)	(0.048)
kessm	0.000	0.000	0.157	0.172	0.206	0.212
	(0.000)	(0.037)	(0.012)	(0.008)	(0.016)	(0.024)
degree	−1.012	−0.680	−0.896	−1.169	−1.191	−0.837
	(0.061)	(0.110)	(0.142)	(0.100)	(0.157)	(0.144)
GCSE	−1.012	−0.680	−0.708	−0.721	−0.789	−0.558
	(0.061)	(0.116)	(0.120)	(0.091)	(0.169)	(0.159)
white	−0.737	0.009	−0.450	−0.580	−0.564	−0.388
	(0.133)	(0.153)	(0.114)	(0.094)	(0.138)	(0.149)
male	−0.000	0.000	0.098	0.184	0.145	0.116
	(0.000)	(0.010)	(0.062)	(0.057)	(0.075)	(0.107)
imdscore	−0.000	0.000	−0.030	−0.060	−0.028	−0.042
	(0.000)	(0.008)	(0.014)	(0.012)	(0.019)	(0.022)
Eng eth stratum	−0.274	0.009	0.216	0.280	0.539	0.805
	(0.116)	(0.056)	(0.115)	(0.118)	(0.151)	(0.133)
Eng dis stratum	−0.000	0.000	0.054	0.130	0.151	0.247
	(0.000)	(0.014)	(0.062)	(0.075)	(0.094)	(0.145)
$\sigma_{\gamma_{q}}^{2}$	0.000	0.750	2.286	2.922	3.404	4.034
	—	—	—	—	—	—
$\sigma_{\gamma_{q}}^{2}$ age	0.000	0.000	0.000	0.151	0.234	0.274
	—	—	—	—	—	—

a Point estimates with standard errors in parentheses.

## 6 Simulation study

In this section we present results from a Monte Carlo simulation study that was used for assessing the performance of MQRE regression at *q*=0.5,0.75,0.9. The objective of this simulation study is twofold. First, we assess the ability of MQRE regression to account for the dependence structure in hierarchical data and hence provide better efficiency compared with models that ignore this dependence structure. Second, we empirically evaluate the analytic approximations for estimating the standard errors of the model parameters. For both aims, data are generated under the two‐level location–shift model

$$y_{ij}=100+2x_{1ij}+\gamma_{j}+\varepsilon_{ij},i=1,\ldots ,n_{j},j=1,\ldots ,100,$$

where the group‐specific sample sizes $n_{j}$ vary between 5 and 20, leading to a total sample size of *n*=1259. The values of $x_{1}∼U\left[0,20\right]$ as well as the sample sizes $n_{j}$ are kept constant over the Monte Carlo replications. The level 1 and level 2 error terms $\gamma_{j}$ and $\varepsilon_{ij}$ are independently generated according to four scenarios:

- [*N*,*N*], normal distributions—*γ*∼*N*(0,3) and *ɛ*∼*N*(0,5);
- [*T*,*T*], *t*‐distributions—*γ*∼*t*(3) and *ɛ*∼*t*(3);
- [*N*,Lap], normal and Laplace distributions—*γ*∼*N*(0,3) and *ɛ*∼Laplace(0,scale=1.58), leading to a level 1 variance of 5;
- [*γ*,*ɛ*], outliers in both hierarchical levels generated via a contamination mechanism—*γ*∼*N*(0,3) for *j*=1,…,90, and *γ*∼*N*(0,20) for *j*=91,…,100, and *ɛ*∼0.9 *N*(0,5)+0.1 *N*(0,150).

Each scenario is independently replicated *R*=500 times. Under scenario [*N*,*N*] the assumptions of the random‐effects model (1) are valid. Scenarios [*T*,*T*] and [*N*,Lap] define situations with clear departures from normality whereas [*γ*,*ɛ*] represents a scenario under outlier contamination in both hierarchical levels. Hence, the assumptions of model (1) are also violated in this case. The tuning constant *c* is set to 1.345 for MQRE regression.

Starting with the first aim, we compare the MQRE and the linear MQ regression model (see Section 4) for which we also use the Huber proposal 2 influence function with *c*=1.345. Although both MQRE regression and MQ regression are based on outlier robust estimation methods, we expect that the MQRE regression will perform better than MQ regression when clustering is present. At *q*=0.5, MQRE regression is compared with the LRE model (1). We expect that MQRE regression will perform better than LRE when the normality assumptions are violated. For location parameters other than *q*=0.5 we compare the MQRE with MQ models. In this case we expect that MQRE regression will be superior when outliers and clustering are present. To compare the various methods we mainly focus on the fixed effects parameters. The results for the variance parameters are available from the authors on request. For each regression parameter, performance is assessed with the following measures:

- average relative bias ARB, defined as
  $$\text{ARB}\left(\hat{\theta }\right)=R^{-1}\sum_{r=1}^{R}\frac{{\hat{\theta }}^{\left(r\right)}-\theta }{\theta }\times 100,$$
  where ${\hat{\theta }}^{\left(r\right)}$ is the estimated parameter at quantile *q* for the *r*th replication and *θ* is the corresponding ‘true’ value of this parameter.
- relative efficiencies EFF, defined as
  $$\text{EFF}\left(\hat{\theta }\right)=\frac{S_{\mathrm{model}}^{2}\left(\hat{\theta }\right)}{S_{\mathrm{MQ}}^{2}\left(\hat{\theta }\right)}$$
  where $S^{2}\left(\hat{\theta }\right)=R^{-1}\Sigma_{r=1}^{R}{\left({\hat{\theta }}^{\left(r\right)}-\overset{¯}{\theta }\right)}^{2}$ and $\overset{¯}{\theta }=R^{-1}\Sigma_{r=1}^{R}{\hat{\theta }}^{\left(r\right)}.$ We use MQ regression with *c*=1.345 as a reference because we are mainly interested in assessing the ability of the MQRE to account for the dependence structure of hierarchical data.

Table 8 reports the simulation results for estimators of the fixed effects under the various approaches for *q*=0.5,0.75,0.9. Under the scenario [*N*,*N*] for *q*=0.5, we observe that the estimators of the fixed effects from LRE regression are more efficient than the corresponding estimators from MQ regression. This is expected because LRE regression correctly models the two‐level structure of the data. The estimators of the fixed effects of the LRE are also more efficient than the corresponding estimators from the MQRE model. Under this scenario there is no reason to employ outlier robust estimation, and doing so results in higher variability for the MQRE regression estimators. At *q*=0.75 and *q*=0.9, the estimators of the fixed effects of the MQRE model are more efficient than the corresponding estimators of MQ regression. This demonstrates the ability of MQRE regression to account for the group structure of the data, which is something that is not possible when using MQ regression.

**Table 8:** Values of bias ARB, efficiency EFF and the average of point estimates over simulations of fixed effects under the four data‐generating scenarios and the alternative regression fits: MQRE, MQ and LRE regression at *q* = (0.5,0.75,0.9)^a^

*Method*	${\hat{\beta }}_{0},\;q=0.5$			${\hat{\beta }}_{1},\;q=0.5$			${\hat{\beta }}_{0},\;q=0.75$			${\hat{\beta }}_{1},\;q=0.75$			${\hat{\beta }}_{0},\;q=0.9$			${\hat{\beta }}_{1},\;q=0.9$		
	*ARB*	*EFF*	$\beta_{0}$	*ARB*	*EFF*	$\beta_{1}$	*ARB*	*EFF*	$\beta_{0}$	*ARB*	*EFF*	$\beta_{1}$	*ARB*	*EFF*	$\beta_{0}$	*ARB*	*EFF*	$\beta_{1}$
*Scenario 1*—[*N*,*N*]																		
MQRE	−0.000	0.807	100.000	−0.013	0.655	2.000	−0.189	0.824	101.316	−0.012	0.696	2.000	−0.349	0.849	102.506	−0.017	0.762	2.000
MQ	0.006	1.000	100.006	−0.034	1.000	1.999	−0.185	1.000	101.320	−0.028	1.000	1.999	−0.347	1.000	102.509	−0.030	1.000	1.999
LRE	−0.001	0.774	99.999	−0.016	0.627	2.000	—	—	—	—	—	—	—	—	—	—	—	—
*Scenario 2*—[*T*,*T*]																		
MQRE	0.002	0.827	100.002	−0.018	0.602	2.000	0.144	0.831	100.910	−0.011	0.607	2.000	0.230	0.838	101.871	−0.005	0.584	2.000
MQ	0.004	1.000	100.004	−0.017	1.000	2.000	0.144	1.000	100.910	−0.006	1.000	2.000	0.227	1.000	101.869	0.005	1.000	2.000
LRE	0.001	1.203	100.001	−0.008	0.824	2.000	—	—	—	—	—	—	—	—	—	—	—	—
*Scenario 3*—[*N*,*Lap*]																		
MQRE	0.011	0.857	100.011	−0.050	0.718	1.999	0.165	0.915	101.262	−0.051	0.817	1.999	−0.073	0.960	102.468	−0.039	0.898	1.999
MQ	0.004	1.000	100.004	−0.024	1.000	2.000	0.157	1.000	101.254	−0.024	1.000	2.000	−0.081	1.000	102.460	−0.018	1.000	2.000
LRE	0.009	0.895	100.009	−0.035	0.784	1.999	—	—	—	—	—	—	—	—	—	—	—	—
*Scenario 4*—[*γ*,*ɛ*]																		
MQRE	−0.004	0.810	99.996	0.025	0.685	2.000	0.126	0.825	101.636	−0.011	0.752	2.000	0.613	0.905	103.496	0.002	0.925	2.000
MQ	−0.008	1.000	99.992	0.030	1.000	2.001	0.121	1.000	101.631	−0.006	1.000	2.000	0.607	1.000	103.490	0.003	1.000	2.000
LRE	−0.005	1.278	99.995	0.042	1.659	2.001	—	—	—	—	—	—	—	—	—	—	—	—

a The results are based on *R*=500 Monte Carlo replications for each of the four scenarios.

The superior performance of MQRE regression is demonstrated in scenarios [*T*,*T*] where the data are generated under a *t*‐distribution and [*ɛ*,*γ*] where outliers exist at both hierarchical levels. In particular, in most cases the estimators of the fixed effects from MQRE regression are more efficient than the corresponding estimators from MQ or from LRE regression. These results provide evidence that using MQRE regression protects against outlying values and accounts for the dependence structure. Finally, it appears that having departures from normality by using a Laplace distribution (scenario [*N*,Lap]) does not have a severe effect on the efficiency of the estimators of the fixed effects in terms of robustness. Nevertheless, we also observe in this scenario a clear advantage of MQRE compared with MQ regression.

Taking a closer look at ARB for the fixed effects, we observe that all estimation methods have almost no bias. The bias is computed by assuming that the target population parameters are the quantiles of the conditional distribution. Hence, the MQRE fits are penalized. Despite this, Table 8 reveals that for *q*=0.5 ARB for the slope and intercept is always smaller than 0.1% for all estimators. The same holds also for the slope at quantile *q*=0.75 and *q*=0.9. In the case of the intercept, ARB is in some cases around 0.3% for *q*=0.75 and around 0.6% for *q*=0.9.

Having assessed the performance of MQRE regression, the second aim of this empirical study is to evaluate the analytic approximations of the standard errors of the fixed effects. Therefore, we compare the empirical and estimated standard errors under the four scenarios. For each scenario and for each estimator $\hat{\theta }$, at *q*=0.5,0.75,0.9, Table 9 reports averages over simulations of the Monte Carlo standard error $S\left(\hat{\theta }\right)=\surd \left\{R^{-1}\Sigma_{r=1}^{R}{\left({\hat{\theta }}^{\left(r\right)}-\overset{¯}{\theta }\right)}^{2}\right\}$ and the estimated standard errors of the fixed effects $\beta_{q}$. It can be observed that for all scenarios the estimated standard error of the estimators at *q*=0.5,0.75,0.9 offers a good approximation to the empirical standard error. Furthermore, for all scenarios the asymptotic standard error of the MQRE regression is, as expected, larger for location parameters that are closer to the tail of the distribution than for location parameters that are closer to the centre of the distribution.

**Table 9:** Empirical standard errors and estimated standard errors of ${\hat{\beta }}_{\psi q}$ for *q*=(0.5,0.75,0.9) using MQRE regression with tuning constant *c*=1.345^a^

*Scenario*	${\hat{\beta }}_{0}$		${\hat{\beta }}_{1}$	
	*Empirical standard error*	*Estimated standard error*	*Empirical standard error*	*Estimated standard error*
*q*=0.5				
1—[*N*,*N*]	0.2219	0.2189	0.0118	0.0116
2—[*T*,*T*]	0.1616	0.1567	0.0072	0.0072
3—[*N*,Lap]	0.2060	0.2127	0.0108	0.0105
4—[*γ*,*ɛ*]	0.2635	0.2611	0.0143	0.0143
*q*=0.75				
1—[*N*,*N*]	0.2340	0.2288	0.0128	0.0126
2—[*T*,*T*]	0.1850	0.1806	0.0089	0.0084
3—[*N*,Lap]	0.2212	0.2263	0.0127	0.0121
4—[*γ*,*ɛ*]	0.2922	0.2962	0.0169	0.0172
*q*=0.9				
1—[*N*,*N*]	0.2624	0.2559	0.0153	0.0152
2—[*T*,*T*]	0.2687	0.2649	0.0133	0.0129
3—[*N*,Lap]	0.2725	0.2715	0.0180	0.0171
4—[*γ*,*ɛ*]	0.4814	0.4693	0.0326	0.0325

a The results are based on *R*=500 Monte Carlo replications for each scenario.

## 7 Discussion

The paper offers to the prospective data analyst tools for modelling a general set of location parameters in the presence of clustering in the data. In particular, we propose an extension of MQ regression to MQRE regression.

As illustrated in the real data example, the proposed approach to modelling conditional MQs can offer a considerably deeper insight to child psychologists into the effect of risk factors on children's behavioural problems. This in turn can assist in proposing new substantive theory. The use of the methodology is facilitated by the availability of a computationally efficient algorithm using C++ in R. The current methodology allows only for random‐intercepts and two‐level structures. Future work will extend the proposed methodology to allow for additional hierarchical levels and more complex correlation structures that include random coefficients.

## Appendix A

The steps of the estimation algorithm are as follows:

*Step 1*: start by assuming that $\left(\sigma_{\gamma_{q}}^{2},\sigma_{\epsilon_{q}}^{2}\right)$ are known.
*Step 2*: given these variance parameters, form the covariance matrix $V_{q}$, and estimate $\beta_{\psi q}$ by solving the iterative equation
  $$\beta_{\psi q}^{t+1}=\beta_{\psi q}^{t}+{\left\{X^{T}U_{q}^{-1/2}D_{q}\left(\beta_{\psi q}^{t}\right)U_{q}^{1/2}V_{q}^{-1}X\right\}}^{-1}X^{T}V_{q}^{-1}U_{q}^{1/2}\psi_{q}\left(r_{q}\right),$$
  where $D_{q}\left(\beta_{\psi q}^{t}\right)$ is a diagonal matrix with *j*th diagonal element $D_{ijq}=\psi_{q}^{\prime }\left(r_{ijq}\right)=\partial \psi_{q}\left(r_{ijq}\right)/\partial r_{ijq}.$
*Step 3*: use the estimates of $\beta_{\psi q}$ to obtain estimates of the variance parameters. The estimates of the variance parameters are obtained by using a fixed point iterative method. This requires us to change the estimating equations (9) to
  $$\begin{array}{cc} & \psi_{q}{\left(r_{q}\right)}^{T}U_{q}^{1/2}V_{q}^{-1}ZZ^{{}^{T}}V_{q}^{-1}U_{q}^{1/2}\psi_{q}\left(r_{q}\right)-K_{2q}\text{tr}\;\left\{V_{q}^{-1}ZZ^{{}^{T}}V_{q}^{-1}\left(ZZ^{{}^{T}}\;I_{n}\right)\;\;\left(\begin{array}{c} \sigma_{\gamma_{q}}^{2}\\ \sigma_{\epsilon_{q}}^{2} \end{array}\right)\right\}=0,\\ & \psi_{q}{\left(r_{q}\right)}^{T}U_{q}^{1/2}V_{q}^{-1}I_{n}V_{q}^{-1}U_{q}^{1/2}\psi_{q}\left(r_{q}\right)-K_{2q}\text{tr}\;\left\{V_{q}^{-1}I_{n}V_{q}^{-1}\left(ZZ^{{}^{T}}\;I_{n}\right)\;\;\left(\begin{array}{c} \sigma_{\gamma_{q}}^{2}\\ \sigma_{\epsilon_{q}}^{2} \end{array}\right)\right\}=0, \end{array}$$
  by replacing $V_{q}$ by $V_{q}=\sigma_{\epsilon_{q}}^{2}I_{n}+\sigma_{\gamma_{q}}^{2}ZZ^{{}^{T}}$ and using $V_{q}^{-1}V_{q}=I_{n}$. The fixed point algorithm of the estimating equations for the *t*th iteration can be presented as follows:
  (11) $$\left(\begin{array}{c} \sigma_{\gamma_{q}}^{2(t+1)}\\ \sigma_{\epsilon_{q}}^{2(t+1)} \end{array}\right)={\left\{A\left(\begin{array}{c} \sigma_{\gamma_{q}}^{2(t)}\\ \sigma_{\epsilon_{q}}^{2(t)} \end{array}\right)\right\}}^{-1}a\left(\begin{array}{c} \sigma_{\gamma_{q}}^{2(t)}\\ \sigma_{\epsilon_{q}}^{2(t)} \end{array}\right)\;\;,$$
  where
  $$A\left(\begin{array}{c} \sigma_{\gamma_{q}}^{2}\\ \sigma_{\epsilon_{q}}^{2} \end{array}\right)=\left(\begin{array}{cc} K_{2q}\text{tr}\left(V_{q}^{-1}ZZ^{{}^{T}}V_{q}^{-1}ZZ^{{}^{T}}\right) & K_{2q}\text{tr}\left(V_{q}^{-1}ZZ^{{}^{T}}V_{q}^{-1}I_{n}\right)\\ K_{2q}\text{tr}\left(V_{q}^{-1}I_{n}V_{q}^{-1}ZZ^{{}^{T}}\right) & K_{2q}\text{tr}\left(V_{q}^{-1}I_{n}V_{q}^{-1}I_{n}\right) \end{array}\right)$$
  and
  $$a\left(\begin{array}{c} \sigma_{\gamma_{q}}^{2}\\ \sigma_{\epsilon_{q}}^{2} \end{array}\right)=(\begin{array}{c} \frac{1}{2}\psi_{q}{\left(r_{q}\right)}^{T}U_{q}^{1/2}V_{q}^{-1}ZZ^{{}^{T}}V_{q}^{-1}U_{q}^{1/2}\psi_{q}\left(r_{q}\right)\\ \frac{1}{2}\psi_{q}{\left(r_{q}\right)}^{T}U_{q}^{1/2}V_{q}^{-1}I_{n}V_{q}^{-1}U_{q}^{1/2}\psi_{q}\left(r_{q}\right) \end{array})\;.$$
  Iterative equation (11) is more stable than the Newton–Raphson method. Like any other iterative algorithm, the fixed point algorithm requires initial values for the parameters. As a result, using well‐defined starting values for the variance parameters is advisable.
*Step 4*: iterate steps 2 and 3 until convergence.
*Step 5*: at convergence, predicted random effects at the *q*th MQ fit are obtained by solving the following estimating equation with respect to $\gamma_{q}$:
  (12) $$Z^{T}\Sigma_{\epsilon_{q}}^{-1/2}\psi_{q}\left\{\Sigma_{\epsilon_{q}}^{-1/2}\left(y-X\beta_{\psi q}-Z\gamma_{q}\right)\right\}-\Sigma_{\gamma_{q}}^{-1/2}\psi_{q}\left(\Sigma_{\gamma_{q}}^{-1/2}\gamma_{q}\right)=0.$$

As can be seen from the steps of the estimation algorithm, predicted random effects are obtained at convergence, i.e. we start by first estimating the fixed effects and the variance parameters and then, given estimates of the fixed effects and of the variance parameters, we predict the random effects. The reason for this, as also pointed out by Sinha and Rao (2009), is that estimates of the variance parameters that depend on the predicted random effects may be unstable.

## Appendix B

The estimators ${\hat{\theta }}_{q}={\left({\hat{\beta }}_{q}^{T},{\hat{\sigma }}_{\gamma_{q}}^{2},{\hat{\sigma }}_{\epsilon_{q}}^{2}\right)}^{T}$ satisfy a set of estimating equations of the form

(13) $$\sum_{j=1}^{d}\Phi_{qj}\left(\theta_{q}\right)=0,$$

where $\Phi_{qj}\left(\theta_{q}\right)={\left(\Phi_{qj\beta_{\psi q}}^{T},\Phi_{qj\sigma_{\gamma_{q}}^{2}},\Phi_{qj\sigma_{\epsilon_{q}}^{2}}\right)}^{T}$, for particular choices of $\Phi_{qj}\left(\theta_{q}\right)$. Under a general response distribution *D*, the estimator ${\hat{\theta }}_{q}$ satisfying equations (12) is estimating a root $\theta_{q}$ of

(14) $$\sum_{j=1}^{d}E_{D}\left[\Phi_{qj}\left(\theta_{q}\right)\right]=0.$$

Under the assumption that the function $\Phi_{qj}\left(\theta_{q}\right)$ is a bounded and non‐decreasing function and has bounded first‐order derivatives and provided that

(15) $$-n^{-1}\sum_{j=1}^{d}E_{D}\left[\partial \Phi_{qj}\left(\theta_{q}\right)/\partial \theta_{q}\right]\to G,$$

where the (*p*+2)×(*p*+2) matrix **G** is positive definite, and

(16) $$n^{-1}\sum_{j=1}^{d}E_{D}\left[\Phi_{qj}{\left(\theta_{q}\right)}^{T}\Phi_{qj}\left(\theta_{q}\right)\right]\to F,$$

a Taylor series approximation which holds uniformly in a neighbourhood of $\theta_{q}$ is

(17) $${\hat{\theta }}_{q}\approx \theta_{q}+G^{-1}n^{-1}\sum_{j=1}^{d}\Phi_{qj}\left(\theta_{q}\right)+o_{p}\left(n^{-1/2}\right),\;\text{as}\;n\to \infty .$$

Following Welsh and Richardson (1997), the covariance matrix of the estimators can be written as $G^{-1}F{\left(G^{-1}\right)}^{T}$. The components of the matrix **G** and the matrix **F** are available from the authors on request. The covariance matrix of the estimators can be consistently estimated by ${\hat{G}}^{-1}\hat{F}{\left({\hat{G}}^{-1}\right)}^{T}$ where the matrices $\hat{G}$ and $\hat{F}$ are evaluated at ${\hat{\theta }}_{q}$.

## References

[rssa12126-bib-0001] Anderson, T. W. (1973) Asymptotically efficient estimation of covariance matrices with linear covariance structure. Ann. Statist., 1, 135–141.

[rssa12126-bib-0002] Bradley, R. H. and Corwyn, R. F. (2002) Socioeconomic status and child development. A. Rev. Psychol., 53, 371–399. 10.1146/annurev.psych.53.100901.135233 11752490

[rssa12126-bib-0003] Breckling, J. and Chambers, R. (1988) *M*‐quantiles. Biometrika, 75, 761–771.

[rssa12126-bib-0004] Chambers, R. and Tzavidis, N. (2006) *M*‐quantile models for small area estimation. Biometrika, 93, 255–268.

[rssa12126-bib-0005] Costello, E. J. , Compton, S. N. , Keeler, G. and Angold, A. (2003) Relationships between poverty and psychopathology: a natural experiment. J. Am. Med. Ass., 290, 2023–2029. 10.1001/jama.290.15.2023 14559956

[rssa12126-bib-0006] Dearing, E. , McCartney, K. and Taylor, B. A. (2006) Within‐child associations between family income and externalizing and internalizing problems. Devlpmntl Psychol., 42, 237–252. 10.1037/0012-1649.42.2.237 16569163

[rssa12126-bib-0007] Fellner, W. H. (1986) Robust estimation of variance components. Technometrics, 28, 51–60.

[rssa12126-bib-0009] Flouri, E. , Midouhas, E. and Joshi, H. (2014a) Family poverty and trajectories of children's emotional and behavioural problems: the moderating roles of self‐regulation and verbal cognitive ability. J. Abnrml Chld Psychol., 42, 1043–1056. 10.1007/s10802-013-9848-3 24473936

[rssa12126-bib-0010] Flouri, E. , Midouhas, E. , Joshi, H. and Tzavidis, N. (2014b) Emotional and behavioural resilience to multiple risk exposure in early life: the role of parenting. Eur. Chld Adolesc. Psychiatr., to be published. 10.1007/s00787-014-0619-7 25300919

[rssa12126-bib-0008] Flouri, E. , Tzavidis, N. and Kallis, C. (2010) Area and family effects on the psychopathology of the Millennium Cohort Study children and their older siblings. J. Chld Psychol. Psychiatr., 51, 152–161. 10.1111/j.1469-7610.2009.02156.x 19804382

[rssa12126-bib-0011] Geraci, M. and Bottai, M. (2007) Quantile regression for longitudinal data using the asymmetric Laplace distribution. Biostatistics, 8, 140–154. 16636139 10.1093/biostatistics/kxj039

[rssa12126-bib-0012] Geraci, M. and Bottai, M. (2014) Linear quantile mixed models. Statist. Comput., 24, 461–479.

[rssa12126-bib-0013] Goodman, R. (1997) The Strengths and Difficulties Questionnaire: a research note. J. Chld Psychol. Psychiatr., 38, 581–586. 10.1111/j.1469-7610.1997.tb01545.x 9255702

[rssa12126-bib-0014] Goodman, S. H. and Gotlib, I. H. (1999) Risk for psychopathology in the children of depressed mothers: a developmental model for understanding mechanisms of transmission. Psychol. Rev., 106, 458–490. 10467895 10.1037/0033-295x.106.3.458

[rssa12126-bib-0015] Goodnight, J. A. , Lahey, B. B. , Van Hulle, C. A. , Rodgers, J. L. , Rathouz, P. J. , Waldman, I. D. and D'Onofrio, B. M. (2012) A quasi‐experimental analysis of the influence of neighborhood disadvantage on child and adolescent conduct problems. J. Abnrml Psychol., 121, 95–108. 10.1037/a0025078 PMC3287068 21942334

[rssa12126-bib-0016] Hartley, H. O. and Rao, J. N. K. (1967) Maximum‐likelihood estimation for the mixed analysis of variance model. Biometrika, 54, 93–108. 6049561

[rssa12126-bib-0017] Huber, P. J. (1967) The behavior of maximum likelihood estimates under nonstandard conditions In Proc. 5th Berkeley Symp. Mathematical Statistics and Probability, vol. , pp. 221–233. Berkeley: University of California Press.

[rssa12126-bib-0018] Huber, P. J. (1981) Robust Statistics. New York: Wiley.

[rssa12126-bib-0019] Huggins, R. M. (1993) A robust approach to the analysis of repeated measures. Biometrics, 49, 255–268.

[rssa12126-bib-0020] Kiernan, K. E. and Huerta, M. C. (2008) Economic deprivation, maternal depression, parenting and children's cognitive and emotional development in early childhood. Br. J. Sociol., 59, 783–806. 19035922 10.1111/j.1468-4446.2008.00219.x

[rssa12126-bib-0021] Koenker, R. (2004) Quantile regression for longitudinal data. J. Multiv. Anal., 91, 74–89.

[rssa12126-bib-0022] Koenker, R. and Bassett, G. (1978) Regression quantiles. Econometrica, 46, 33–55.

[rssa12126-bib-0024] Kokic, P. , Chambers, R. , Breckling, J. and Beare, S. (1997) A measure of production performance. J. Bus. Econ. Statist., 10, 419–435.

[rssa12126-bib-0025] Midouhas, E. , Kuang, Y. and Flouri, E. (2014) Neighbourhood human capital and the development of children's emotional and behavioural problems: the mediating role of parenting and schools. Hlth Place, 27, 155–161. 10.1016/j.healthplace.2014.02.004 24607874

[rssa12126-bib-0026] Miller, J. J. (1977) Asymptotic properties of maximum likelihood estimates in the mixed model analysis of variance. Ann. Statist., 5, 746–762.

[rssa12126-bib-0027] Newey, W. K. and Powell, J. L. (1987) Asymmetric least squares estimation and testing. Econometrica, 55, 819–847.

[rssa12126-bib-0028] Plomin, R. , DeFries, J. C. , McClearn, G. E. and Rutter, M. (1997) Behavioral Genetics: Primer, 3rd edn. New York: Freeman.

[rssa12126-bib-0029] R Development Core Team (2010) R: a Language and Environment for Statistical Computing. Vienna: R Foundation for Statistical Computing.

[rssa12126-bib-0030] Richardson, A. M. and Welsh, A. H. (1995) Robust estimation in the mixed linear model. Biometrics, 51, 1429–1439.

[rssa12126-bib-0031] Silberg, J. L. , Maes, H. and Eaves, L. J. (2010) Genetic and environmental influences on the transmission of parental depression to children's depression and conduct disturbance: an extended Children of Twins study. J. Chld Psychol. Psychiatr., 51, 734–744. 10.1111/j.1469-7610.2010.02205.x PMC2891390 20163497

[rssa12126-bib-0032] Sinha, S. K. and Rao, J. N. K. (2009) Robust small area estimation. Can. J. Statist., 37, 381–399.

[rssa12126-bib-0034] Staudenmayer, J. , Lake, E. E. and Wand, M. P. (2009) Robustness for general design mixed models using the t‐distribution. Statist. Modllng, 9, 235–255.

[rssa12126-bib-0033] Steele, F. (2008) Multilevel models for longitudinal data. J. R. Statist. Soc. A, 171, 5–19.

[rssa12126-bib-0035] Tiet, Q. Q. , Bird, H. R. , Davies, M. , Hoven, C. , Cohen, P. , Jensen, P. S. and Goodman, S. (1998) Adverse life events and resilience. J. Am. Acad. Chld Adolesc. Psychiatr., 37, 1191–1200. 10.1097/00004583-199811000-00020 9808931

[rssa12126-bib-0036] Trentacosta, C. J. , Hyde, L. W. , Shaw, D. S. , Dishion, T. J. , Gardner, F. and Wilson, M. (2008) The relations among cumulative risk, parenting, and behavior problems during early childhood. J. Chld Psychol. Psychiatr., 49, 1211–1219. 10.1111/j.1469-7610.2008.01941.x PMC2683369 18665880

[rssa12126-bib-0037] Venables, W. N. and Ripley, B. D. (2002) Modern Applied Statistics with S. New York: Springer.

[rssa12126-bib-0038] Welsh, A. H. and Richardson, A. M. (1997) Approaches to the robust estimation of mixed models In Handbook of Statistics, vol. (eds Maddala G. S. and Rao C. R.), ch. 13. Amsterdam: Elsevier.

